# Twenty-year trends in racial and ethnic enrollment in large diabetes randomized controlled trials

**DOI:** 10.1186/s12916-022-02501-2

**Published:** 2022-09-16

**Authors:** Jingyi Zhang, Harriette G. C. Van Spall, Yaoyao Wang, Lehana Thabane, Ruoting Wang, Guowei Li

**Affiliations:** 1 Center for Clinical Epidemiology and Methodology (CCEM), Guangdong Second Provincial General Hospital, Guangzhou, China; 2 Department of Health Research Methods, Evidence, and Impact (HEI), McMaster University, Hamilton, ON Canada; 3 Department of Medicine, McMaster University, Hamilton, ON Canada; 4 Department of Epidemiology, School of Medicine, Jinan University, Guangzhou, China; 5 St Joseph’s Healthcare Hamilton, ON Hamilton, Canada; 6 Faculty of Health Sciences, University of Johannesburg, Johannesburg, South Africa

**Keywords:** BIPOC, Enrollment, Diabetes, Randomized controlled trials

## Abstract

**Background:**

Lack of representativeness in Black, Indigenous, and People of Colour (BIPOC) enrollment could compromise the generalizability of study results and health equity. This study aimed to examine trends in BIPOC groups enrollment in diabetes randomized controlled trials (RCTs) and to explore the association between trial factors and high-enrollment of BIPOC groups.

**Methods:**

We systematically searched the literature on large diabetes RCTs with a sample size of ≥ 400 participants published between 2000 and 2020. We assessed temporal trends in enrollment of racial and ethnic groups in the included trials. Logistic and linear regression analyses were used to explore the relationship between trial factors and the high-enrollment defined by median enrollment rate.

**Results:**

A total of 405 RCTs were included for analyses. The median enrollment rate of BIPOC groups was 24.0%, with 6.4% for the Black group, 11.2% for Hispanic, 8.5% for Asian, and 3.0% for other BIPOC groups respectively. Over the past 20 years, the BIPOC enrollment showed an increased trend in the diabetes RCTs, ranging from 20.1 to 28.4% (*P* for trend = 0.041). A significant trend towards increased enrollment for Asian group was observed. We found that weekly or daily intervention frequency (OR = 0.48, 95% CI: 0.26, 0.91) and duration of intervention > 6.5 month (OR = 0.59, 95% CI: 0.37, 0.95) were significantly related to decreased odds of high-enrollment, while type 2 diabetes (OR = 1.44, 95% CI: 1.04, 1.99) was associated with high-enrollment of BIPOC groups.

**Conclusions:**

The enrollment of BIPOC was found to increase in large diabetes RCTs over the past two decades; some trial factors may be significantly associated with BIPOC enrollment. These findings may highlight the importance of enrollment of BIPOC groups and provide insights into the design and implementation of future clinical trials in diabetes.

**Supplementary Information:**

The online version contains supplementary material available at 10.1186/s12916-022-02501-2.

## Background

The prevalence of diabetes substantially varies by racial and ethnic groups [1–3], for instance, the prevalence of diabetes was significantly higher in Black, Indigenous, and People of Colour (BIPOC) groups compared with the White participants in the USA and UK [4–8]. The BIPOC participants generally had a lower rate of diabetes diagnosis and required more attention for the quality of treatment and care, because of their socioeconomic status, health insurance, education level, religious beliefs, and language barriers, among others [9–12]. While the majority of participants were White groups in clinical trials especially from the western countries [13], approximately one fifth of approved new drugs showed differences in drug exposure and response across racial and ethnic groups, with only a few cases translated into racial- and ethnicity-specific treatment recommendations [14]. Therefore, lack of representativeness in BIPOC enrollment could compromise the generalizability of study results and health equity [15].

According to the National Institutes of Health (NIH) [16, 17], adequate enrollment and analysis plans related to racial and ethnic groups are essentially needed in clinical research, where the adequate enrollment could be determined by using the participation to prevalence ratio that is the percentage of BIPOC among trial participants divided by the percentage of BIPOC in the overall diabetes population [8]. Despite the importance of representative racial and ethnic enrollment, the current practice remained suboptimal in many randomized controlled trials (RCTs) including trials of cancer [18–20], stroke [21], systemic lupus erythematosus [22], cardiovascular disease [23–27], obesity [28], acute pain [29], coronavirus disease 2019 [30], and vaccine [31]. Likewise, one previously study reported that there were 62% and 78% of the diabetes RCTs conducted in the US and UK respectively under-enrolling the BIPOC groups [8]. However, there was an evidence gap in diabetes RCTs regarding the current status of and temporal trend in the overall enrollment of BIPOC groups.

Therefore, in this study, we aimed to examine the status of and temporal trend in enrollment of BIPOC groups in large diabetes RCTs with a sample size of ≥ 400 participants. We also explored the potential relationship between trial factors and high-enrollment of BIPOC groups. The research protocol of this study was registered in PROSPERO (International Prospective Register of Systematic Reviews, CRD42021229100).

## Methods

### Search strategy

Details on the study procedures have been published elsewhere [8]. In brief, we comprehensively searched the following electronic databases: the Cochrane Library, MEDLINE (via PubMed), and EMBASE, by using the terms “diabetes mellitus” and “randomized controlled trials.” The search covered the time span from January 1, 2000, to December 31, 2020. We also searched the World Health Organization Clinical Trials Registry Platform, ClinicalTrials, and Google Scholar. In addition, we searched the reference lists of the identified studies for further potential studies. The search strategies were determined by multiple pre-searches; Supplemental Table 1 shows the MEDLINE search strategies.

### Inclusion and exclusion criteria

We included both type 1 and type 2 diabetes RCTs involving multiple (≥ 2) racial and ethnic participants. Other inclusion criteria were participants over the age of 18, and a sample size of at least 400 participants, because trials with smaller a sample size were more likely to be early-stage and single-center studies. There were some publications from different stages of a trial, different subgroup or exploratory analyses; we only included those trials reporting main outcomes with baseline data for the whole population. We excluded trials that focused on gestational diabetes because the type of gestational diabetes may be a temporal form and could theoretically return to normal dependent on glucose control [32, 33]. Those trials that pre-defined some specific BIPOC were also excluded; for instance, the trial exclusively enrolling the Black group was not eligible. We also excluded duplicates, conference abstracts, comments and letters, studies published in languages other than English, and studies with no access to full text and data extraction.

### Study selection

After eliminating duplicates by software and manual check, two reviewers (J Zhang and Y Wang) independently screened and reviewed titles and abstracts retrieved from the search before selecting potentially relevant studies. Subsequently, the two reviewers screened the full texts and determined final selection of trials. A pilot test was conducted before screening the literature to ensure that each reviewer fully understood the inclusion and exclusion criteria and process. Disagreements about the selection of studies were solved by consulting a third reviewer (G Li). The detailed process of study selection was displayed in a flow diagram (Supplemental Fig. 1) [34].

### Outcomes

The primary outcomes were the status of and temporal trend in overall BIPOC enrollment from the included RCTs. The secondary outcomes included the trends in specific BIPOC enrollment that included Black, Asian, Hispanic, and other BIPOC groups.

We also explored the relationship between trial factors (details below) and high-enrollment of BIPOC participants, where the median enrollment rate was used to categorize the included RCTs into either high-enrollment trials or reference trials.

### Data extraction

Two reviewers (J Zhang and Y Wang) independently extracted the following data from included trials using a standardized extraction sheet: (I) percentage enrollment of racial and ethnic group (all BIPOC, Black, Asian, Hispanic, other BIPOC groups), (II) publication information (first and corresponding authors, year of publication), (III) study details (type of RCTs, type of diabetes, type of complication, sample size, age, sex, research purpose, country or region of trial coordination office, outpatient enrollment, source of funding), and (IV) intervention and follow-up details (type of intervention, frequency and duration of intervention, type of follow-up, frequency and duration of follow-up). Disagreements were addressed through discussion with a third reviewer (G Li).

Trial factors for analyses of interest in this study included year of publication, sample size, type of diabetes, enrollment location, type of RCTs, source of funding, the intervention, and follow-up details. Patient age and sex were outcomes of the eligibility criteria or enrollment process, as was race and ethnicity. Therefore, patient age and sex were not included for analyses to explore the relationship with enrollment of BIPOC.

### Statistical analyses

We described continuous variables with median and interquartile range (IQR) and categorical variables with counts and percentages. We used the kernel-weighted local polynomial smoothing curve to present the percentages of the BIPOC enrollment with the ascending year of publication. The Jonckheere-Terpstra proportion trend test was employed to explore whether there was a significant trend for the BIPOC participant enrollment over time.

We used univariate and multivariable logistic regression analyses to analyze the association between trial factors and high-enrollment of BIPOC groups. All the trial factors were included in the multivariable logistic model to retain all possible factors in the model, taking the exploratory nature of our analyses into consideration. To enhance simplicity and interpretability, we used median values to dichotomize the continuous trial factors before they were entered into the model. Odds ratios (ORs) and their corresponding 95% confidence intervals (95% CIs) were employed to quantize the relationship. We also performed a sensitivity analysis by using linear regression analyses to assess the associations between trial factors and the continuous BIPOC enrollment rate, with beta coefficients (*β*s) and 95% CIs showed for the relationship.

All the data analyses were performed with STATA software (version 16.0), and a two-sided *P* value less than 0.05 was considered statistically significant.

## Results

We identified 18,278 records in the initial literature search, among which 1463 literatures were retrieved for full-text review. A total of 405 RCTs were included for our analyses, with over two thirds of RCTs published in the last decade (2010–2020). Among the 405 trials, 84.4% reported data on Black group, 72.8% on Asian, 27.2% on Hispanic, and 89.1% on other BIPOC groups. Table 1 summarizes the factors of the included RCTs. 80.7% of the included RCTs were conducted in multiple countries, and 19.3% were single-country trials. Supplemental Table 2 shows the country or region of trial coordination office for the multi-country RCTs, with 23.2% in North America and 14.7% in Europe. More than two thirds of the trials focused on type 2 diabetes (85%). The included trials had a median sample size of 716 (IQR: 527–1246), female proportion of 46% (IQR: 39.9–50.1%), and age of 58 years old (IQR: 55.0–60.8). The trial aims mainly included glycemic control (31%), management (43%), and diabetic complication (26%). Most of the trials explored the intervention of drugs (86%); and the median intervention duration was 6.5 months (IQR: 6.0–13.5). More than half of the trials were conducted through a face-to-face follow-up; the median duration of follow-up was 12 months (IQR: 6.0–18.0). The majority of the trials received funding form industry (75.9%).

**Table 1:** Basic characteristics of the included diabetes RCTs published between 2000 and 2020

Trial characteristics	Overall RCTs (*n* = 405)
**Year of publication**: *n* (%)	
2000–2004	36 (8.9)
2005–2009	97 (24.0)
2010–2014	135 (33.3)
2015–2020	137 (33.8)
**Multi-country trials:** *n* (%)	327 (80.7)
**Sample size**: median (Q1, Q3)	716 (527, 1246)
**Age**: median (Q1, Q3), years	58 (55.0, 60.8)
**Female proportion**: *n* (%)	46 (39.9, 50.1)
**Trial aim**: *n* (%)	
Glycemic control	125 (30.9)
Management	173 (42.7)
Complication	105 (25.9)
Mixed	2 (0.5)
**Trial reimbursement for patients:** *n* (%)	0
**Outpatient enrolment: *n* (%)**	164 (40.4)
**Type of diabetes**: *n* (%)	
Type 1 diabetes	16 (4.0)
Type 2 diabetes	346 (85.4)
Unspecified	43 (10.6)
**Type of randomization**: *n* (%)	
Individual	399 (98.5)
Cluster	6 (1.5)
**Type of intervention**: *n* (%)	
Drug	347 (85.7)
Lifestyle or education	13 (3.2)
Device	8 (2.0)
Others	37 (9.1)
**Frequency of intervention**: *n* (%)	
> 1 time/week	325 (80.2)
1 ~ 4 times/month	11 (2.7)
> 1 time/ year	3 (0.7)
Not reported	66 (16.4)
**Duration of intervention**: median (Q1, Q3), months	6.5 (6.0, 13.5)
**Type of follow-up**: *n* (%)	
Face-to-face	231 (57.0)
Telephone	11 (2.7)
Others	33 (8.1)
Not reported	130 (32.2)
**Frequency of follow-up**: *n* (%)	
Weekly	6 (1.5)
Monthly	32 (7.9)
Yearly	29 (7.2)
Not reported	338 (83.4)
**Duration of follow-up**: median (Q1, Q3), months	12 (6.0, 18.0)
**Funding source**: *n* (%)	
Public	67 (16.5)
Industry	304 (75.0)
Combination	28 (6.9)
Not reported	6 (1.6)

*RCT* randomized controlled trial, *Q1* first quartile, *Q3* third quartile

### Trends in enrollment proportion for BIPOC over time

**Fig. 1:**
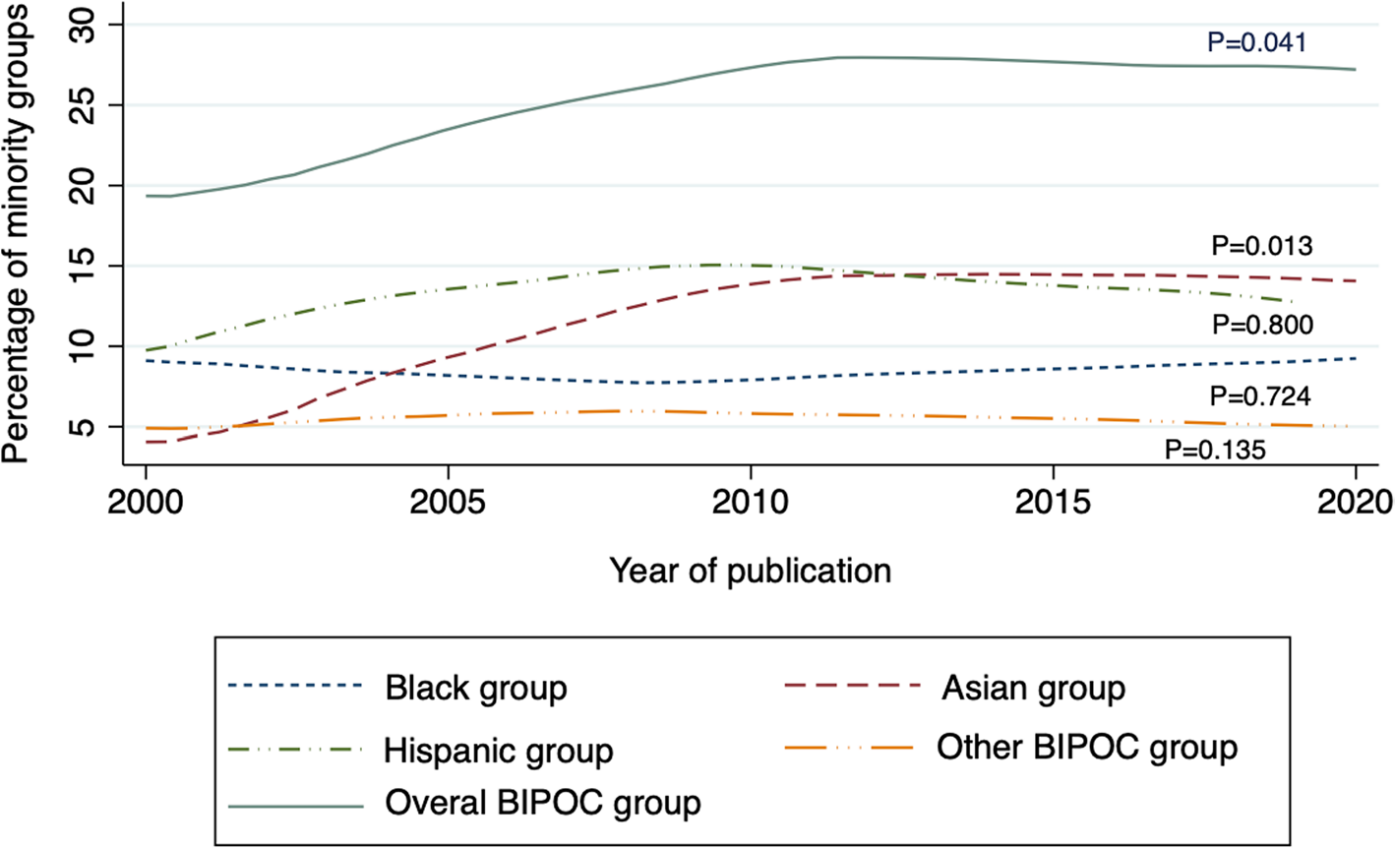
The temporal trends in the BIPOC enrollment between 2000 and 2020

The median enrollment rate of overall BIPOC groups was 24.0% (6.4% for the Black group, 11.2% for Hispanic, 8.5% for Asian, and 3.0% for other BIPOC groups respectively). Figure 1 shows the temporal trends in the BIPOC enrollment. Over the past 20 years, there was a significant trend towards increased BIPOC enrollment in the diabetes trials (*P* for trend = 0.041), with the enrollment ranging from 20.1 to 28.4%. An increased trend in the enrollment of the Asian group was observed (*P* for trend = 0.013), where the enrollment increased from 4.3 to 15.6%. However, no significant trends in enrollment of Hispanic, Black or other BIPOC groups were found.

### Relationship between trial factor and BIPOC high-enrollment

Results from multivariable analyses revealed that weekly or daily intervention frequency (OR = 0.48, 95% CI: 0.26, 0.91) and duration of intervention > 6.5 month (OR = 0.59, 95% CI: 0.37, 0.95) were significantly associated with decreased odds of high-enrollment of overall BIPOC groups, while type 2 diabetes (OR = 1.44, 95% CI: 1.04, 1.99) was significantly related to elevated high-enrollment (Table 2).

**Table 2:** Results from multivariable logistic regression analysis for the relationship between trial characteristics and high-enrollment of BIPOC groups

Trial factors	OR (95%CI)	*P*
Year of publication		
2000–2009	Ref	-
2010–2020	1.75 (0.95, 3.22)	0.074
Sample size ≥ 716^a^	0.98 (0.64, 1.51)	0.944
Diabetes type		
Other	Ref	-
Type 2 diabetes	1.44 (1.04, 1.99)	0.027
Enrollment location		
Other	Ref	-
Ambulatory	0.55 (0.27, 1.12)	0.097
Random type		
Cluster	Ref	-
Individual	1.00 (0.15, 6.69)	0.998
Trial primary objective		
Complication	Ref	-
Glucose control	1.48 (0.75, 2.89)	0.256
Management or mixed	0.86 (0.48, 1.57)	0.628
Intervention		
Type of intervention		
Others	Ref	-
Medication	0.82 (0.38, 1.77)	0.617
Frequency of intervention		
Others	Ref	-
Weekly/daily	0.48 (0.26, 0.91)	0.024
Duration of intervention > 6.5 month^a^	0.59 (0.37, 0.95)	0.030
Follow-up		
Type of follow-up		
Others	Ref	-
Face-to-face	0.71 (0.41, 1.23)	0.221
Frequency of follow-up		
Other	Ref	-
Weekly/monthly	1.28 (0.62, 2.66)	0.503
Duration of follow-up > 12 months^a^	1.45 (0.88, 2.38)	0.147
Funding source		
Non-industry	Ref	-
Industry	0.86 (0.49, 1.53)	0.614

^a^The cut-off point was determined by using the median value

*Ref* reference category/level

**Fig. 2:**
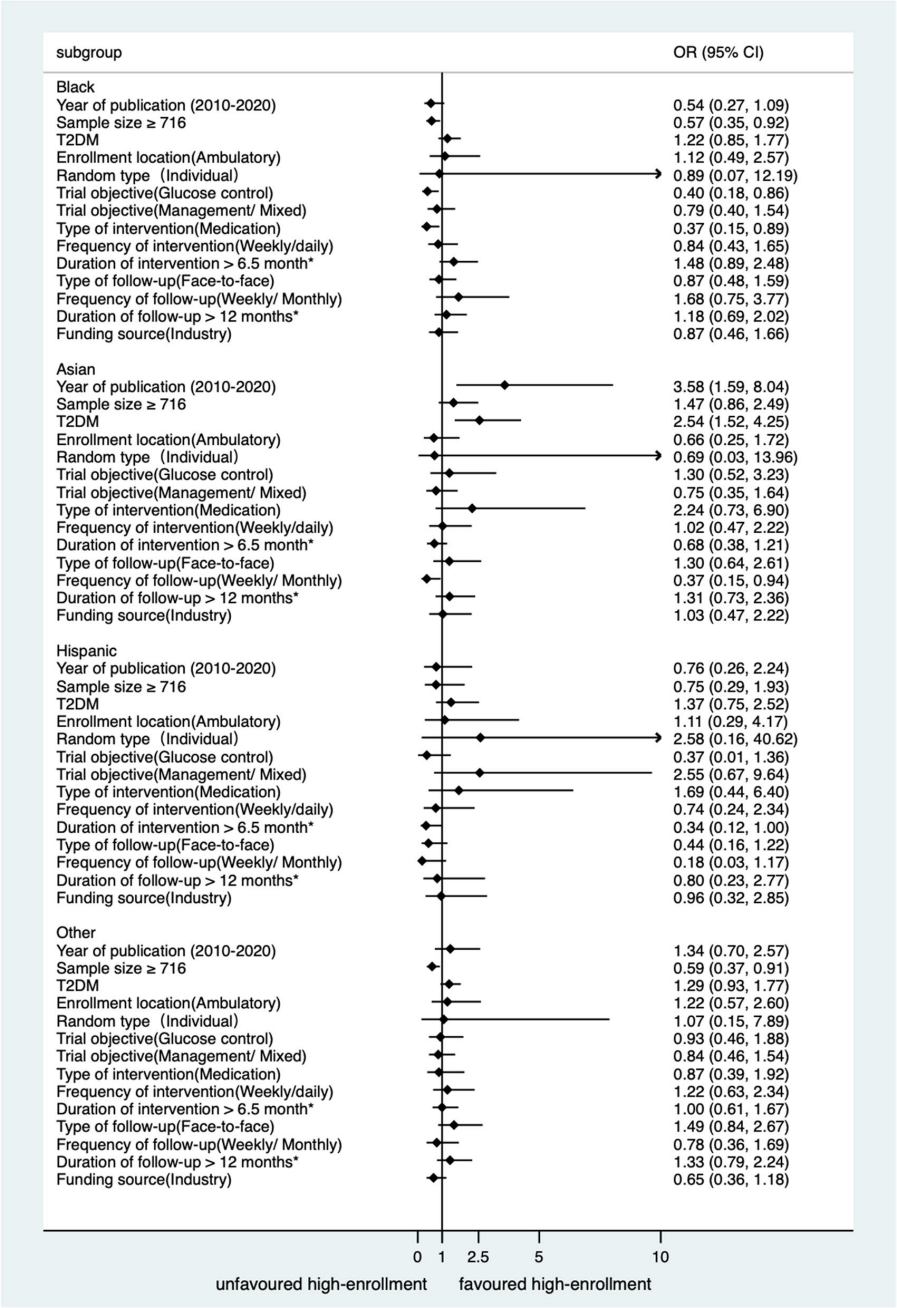
Results from multivariable logistic regression analysis for the relationship between trial characteristics and high-enrollment of BIPOC groups

Supplemental Table 3 and Fig. 2 show univariate and multivariable logistic regression results for the relationship between trial factors and the BIPOC high-enrollment in specific groups. For the Black group, a sample size ≥ 716 (OR = 0.57, 95% CI: 0.35, 0.92), trial objective of glucose control (OR = 0.40, 95% CI: 0.18, 0.86), and intervention of medicine (OR = 0.37, 95% CI: 0.15, 0.89) were significantly related with lower odds of high-enrollment. Regarding Asian group, while weekly or monthly frequency of follow-up (OR = 0.37, 95% CI: 0.15, 0.94) was significantly associated with lower odds of high-enrollment, recent publication year (2010–2020; OR = 3.58, 95% CI: 1.59, 8.04) and type 2 diabetes (OR = 2.54, 95% CI: 1.52, 4.25) were related with increased high-enrollment. Duration of intervention > 6.5 months (OR = 0.34, 95% CI: 0.12, 1.00) was found to significantly associate with decreased odds of high-enrollment of Hispanic group. For other BIPOC groups, those trials with a sample size ≥ 716 (OR = 0.58, 95% CI: 0.37, 0.91) had lower odds of high-enrollment.

### Sensitivity analyses

Findings from sensitivity analyses using linear regression analyses were displayed in Supplemental Table 4, Table 3, and Supplemental Table 5, where the results were largely in line with main analyses from the logistic regression.

**Table 3:** Results from multivariable linear regression analysis for the relationship between trial characteristics and enrollment rate of BIPOC groups

Trial factors	Estimated *β (95%CI)*	*P*
Year of publication		
2000–2009	Ref	-
2010–2020	5.03 (0.31, 9.75)	0.037
Sample size ≥ 716^a^	− 1.82 (− 5.17, 1.54)	0.287
Diabetes type		
Other	Ref	-
Type 2 diabetes	2.59 (0.17, 5.01)	0.036
Enrollment location		
Other	Ref	-
Ambulatory	− 5.60 (− 11.06, − 0.14	0.044
Random type		
Cluster	Ref	-
Individual	− 4.06 (− 18.11, 9.98)	0.570
Trial primary objective		
Complication	Ref	-
Glucose control	3.07 (− 2.06, 8.20)	0.240
Management/ Mixed	− 1.78 (− 6.39, 2.83)	0.449
Intervention		
Type of intervention		
Others	Ref	-
Medication	− 4.48 (− 10.41, 1.44)	0.138
Frequency of intervention		
Others	Ref	-
Weekly/daily	− 1.73 (− 6.49, 3.03)	0.476
Duration of intervention > 6.5 month^a^	− 4.91 (− 8.56, − 1.27)	0.008
Follow-up		
Type of follow-up		
Others	Ref	-
Face-to-face	− 1.80 (− 5.97, 2.38)	0.398
Frequency of follow-up		
Other	Ref	-
Weekly/ Monthly	0.004 (− 5.57, 5.58)	0.999
Duration of follow-up > 12 months^a^	0.07 (− 3.81, 3.96)	0.970
Funding source		
Non-industry	Ref	-
Industry	− 0.42 (− 4.84, 4.00)	0.853

^a^The cut-off point was determined by using the median value

*Ref* reference category/level

## Discussion

In this study, we found that there was a significant trend towards increased overall the BIPOC enrollment in diabetes trials over the past two decades. While an increased trend in the Asian enrollment was detected over time, no significant temporal changes in enrollment of Black, Hispanic, and other BIPOC groups were observed. Some trial factors including type of diabetes, intervention frequency, and duration of intervention were significantly related with high-enrollment of overall BIPOC groups from our exploratory analyses.

The enrollment of overall BIPOC and Asian participants were found to have a significantly increased trend in diabetes RCT. While this phenomenon required further exploration, part of the interpretations may be due to the fact that it reflected the altering overall BIPOC populations in the countries or regions where the trials were conducted. A systematic review demonstrated similar results in heart failure clinical trials; while the trend in the enrollment of Black participants remained stable, Asian (from 1.9 to 10.8%) and Hispanic (5.4 to 14.5%) enrollment showed an increased trend from 2001 to 2018 [35]. A more recent review of HF RCTs confirmed a temporal increase in enrollment of BIPOC groups and reporting of race and ethnicity data, both of which were independently associated with trial leadership by a woman [27]. However, one recent study reported that the enrollment of racial and ethnic groups decreased in clinical research of urate-lowering drugs (from 8.7 to 2.2%) over the past decade [36]. Moreover, one study focusing on trials of anti-cancer drugs for Food and Drug Administration (FDA) approval reported the enrollment of overall racial and ethnic groups remained largely stable over time, with a slight increase among the Hispanic participants and a decline among the Black participants from 2013 to 2018 [18]. From 1985 to 2016, the enrollment of White participants remained dominantly high (over 80%), while the enrollment of Black participants was less than 5% and other BIPOC groups less than 6% in the prostate cancer clinical trials [37]. Therefore, our study indicated that although it may remain suboptimal, the enrollment of BIPOC groups in diabetes trials had improved temporally, especially when compared with gout and cancer trials.

There were no relevant studies that comprehensively analyzed the enrollment of BIPOC groups in diabetes RCTs, to the best of our knowledge. There was only a study with type 1 diabetes describing that the enrollment rates of BIPOC groups were significantly lower than the current prevalence in the USA, in which it included eight studies and focused on FDA-approved technologies from 2015 to 2020 [38]. Other comparable studies with a small sample size may either target other diseases (e.g., cancer [22]), or at specific countries (e.g., the US [28, 39]) or the drugs for FDA approval [38, 40–42], or focused on the NIH-funded trials [43]. While their main findings demonstrated that the enrollment of BIPOC groups remained inadequate, our results generated evidence to the temporal trend and current practice in diabetes RCTs. These findings may provide insights into the design and implementation of future clinical trials in diabetes, especially given the substantial rise of type 2 diabetes among the young people from BIPOC communities.

The BIPOC groups were willing to participate in clinical trials [44]. However there were barriers preventing BIPOC groups from obtaining equitable access to health researches [45]. The racial and ethnic low-enrollment in clinical research was due to several important reasons, including structural racism and socio-economic disadvantage [46, 47]. The implicitly biased perceptions of the BIPOC candidates by physicians or research staff hindered their opportunities to communicate effectively with participants and prevented the BIPOC recruitment in study design [48]. Participants may be less willing to participate in a trial, if the enrollment staff did not have a similar cultural and ethnic background [38]. The family composition, personal relationships with patients and community, investigator and participant training and mentoring, and engagement and operational practices of healthcare professionals also played important roles in BIPOC group enrollment [16, 49, 50]. It is recommended that researchers need to overcome these difficult challenges to enhance the enrollment of BIPOC groups in order to ensure the validity of results and reliable benefits for all.

Several trial factors were found to associate with enrollment rates of BIPOC groups. The frequency of interventions was also associated with reduced enrollment of BIPOC groups. For instance, BIPOC populations may be more likely to encounter barriers in trial recruitment in some behavioral interventions delivered on a weekly or daily basis [15]. By contrast, type 2 diabetes was found to significantly related to elevated high-enrollment. Because type 1 diabetes accounted for a small proportion of diabetes and was common in children and adolescents [51, 52], it would therefore be less likely enroll BIPOC groups in adult trials of type 1 diabetes. Although funding resource was not significantly related to enrollment of BIPOC groups in RCTs of diabetes, it had important implications in studies of other diseases. One recent study reported that the Black enrollment rate in industry-sponsored trials was only a third of the national cancer institute-sponsored trials in Cancer Clinical Trials, mainly because of their biased perceptions of BIPOC groups with low compliance [42]. While the majority of phase III clinical trials were funded through pharmaceutical companies or private sources [53, 54], the low-enrollment of BIPOC groups would remain or even aggravate in trials not funded by public resources. Taken together, although these associations between trial factors and BIPOC group enrollment required further clarification and exploration, our exploratory analysis findings may help with researchers when considering the enrollment of BIPOC participants from the aspects of trial design and implementation.

## Strengths and limitations

Our study was the first to assess the temporal trend in enrollment of BIPOC groups and explore trial factors associated with the enrollment in diabetes RCTs. Our results may highlight the importance of the enrollment of BIPOC groups to the design of future clinical trials. There are several limitations in this research. First, we may have missed some studies in non-English language. Likewise, those trials with unpublished data were not included for our analyses, which may lead to publication bias. Second, we did not have access to enrollment data of BIPOC group for specific countries from the multi-national trials. Therefore, we could not calculate the participation to prevalence ratio for the diabetes population stratified by race and ethnicity to define under-representation of BIPOC groups in specific countries [55, 56]. Third, the BIPOC enrollment may have been affected by reporting bias [25]. The reporting of racial and ethnic enrollment was largely lacking in clinical trials, including the inclusion and exclusion criteria related to BIPOC groups and the enrollment rate of racial and ethnic groups [40, 57]. Therefore, some studies that enrolled BIPOC groups but did not report these data in their literatures were not included in our analyses. Another limitation was that we excluded trials with a sample size < 400 participants for analyses. This may lead to selection bias and thus weaken the strength of our study findings, especially given the fact that the majority of interventions evaluated in large RCTs may be being tested on the minority of the populations who will use them with corresponding citations. Moreover, even though we performed exploratory analyses for trial factors in relation to BIPOC group enrollment, there may be other trial factors that were unable to capture but could bias our findings in the regression analyses.

## Conclusions

The enrollment of BIPOC was found to improve in diabetes RCTs over the past two decades; some trial factors may be significantly associated with BIPOC group enrollment. These findings may highlight the importance of enrollment of BIPOC groups and provide insights into the design and implementation of future clinical trials in diabetes.

## Supplementary Information

**Additional file 1:** **Supplemental Figure 1.** Flow diagram showing the trial selection process. **Supplemental Table 1.** Search strategy used in MEDLINE. **Supplemental Table 2.** Country or region of trial coordination office for the multi-country trials. **Supplemental Table 3.** Results from univariable logistic regression analysis for the relationship between trial characteristics and high-enrollment of BIPOC groups. **Supplemental Table 4.** Results from univariable linear regression analysis for the relationship between trial characteristics and enrollment rate of BIPOC groups. **Supplemental Table 5.** Results from multivariable linear regression analysis for the relationship between trial characteristics and enrollment rate of BIPOC groups

## Data Availability

Not applicable.

## Abbreviations

BIPOC: Black, Indigenous, and People of Colour
RCTs: Randomized controlled trials
NIH: National Institutes of Health
IQR: Interquartile range
ORs: Odds ratios
CI: Confidence interval
*βs*: Beta coefficients
FDA: Food and Drug Administration
