# Supplementary material for: Twenty-year trends in racial and ethnic enrollment in large diabetes randomized controlled trials
Source: BMC Med. 2022 Sep 16;20:294. doi: 10.1186/s12916-022-02501-2 (PMC9479279; doi:10.1186/s12916-022-02501-2)
Supplement: Supplementary file 1 — Additional file 1: Supplemental Figure 1. Flow diagram showing the trial selection process. Supplemental Table 1. Search strategy used in MEDLINE. Supplemental Table 2. Country or region of trial coordination office for the multi-country trials. Supplemental Table 3. Results from univariable logistic regression analysis for the relationship between trial characteristics and high-enrollment of BIPOC groups. Supplemental Table 4. Results from univariable linear regression analysis for the relationship between trial characteristics and enrollment rate of BIPOC groups. Supplemental Table 5. Results from multivariable linear regression analysis for the relationship between trial characteristics and enrollment rate of BIPOC groups [file 12916_2022_2501_MOESM1_ESM.docx]

**Supplemental figure 1.** Flow diagram showing the trial selection process

Records identified through database searching (n=18278)

- PubMed (n=15644)
- EMBASE (n=2634)

Records after duplicates removed
(n=17706)

Records screened
(n=17706)

Records excluded (n =16243)

- Irrelevant (n=15268)
- Sample size of < 400 (n=774)
- Children (n=128)
- Post hoc text/Secondary analyses/Summary analyses (n=24)
- Abstract/Letter/News/Comment (n=18)
- Duplicate (n=15)
- Pregnancy(n=13)
- Animal (n=3)

Full-text articles assessed for eligibility
(n=1463)

Studies included in RCT (n=405)

Full-text articles excluded (n=1058)

- Non- ethnical data (n=456)
- Post hoc text/Secondary analyses/Summary analyses (n=225)
- Trial not including diabetic patients and effect of intervention on diabetes (n=190)
- Lack of sufficient data (n=101)
- Non-RCT (n=32)
- Sample size of < 400 (n=21)
- Trial targeting a specific minor race (n=15)
- Duplicates (n =13)
- Non-English (n=5)

## Identification

**Screening**

**Eligibility**

**Included**

**Supplemental table 1.** Search strategy used in MEDLINE

| **Search steps** | **Search terms** |
| --- | --- |
| 1 | adult[MeSH Terms] |
| 2 | "diabetes mellitus"[MeSH Terms] |
| 3 | "Diabetes, Gestational"[Mesh] |
| 4 | "Randomized Controlled Trials as Topic"[Mesh] |
| 5 | randomized controlled trial"[Publication Type] |
| 6 | 2 not 3 |
| 7 | 4 or 5 |
| 8 | 1 and 6 and 7 |
| 9 | limit 8 to (English language or humans) |
| 10 | limit 9 to yr=*2000-2020* |

**Supplemental table 2. Country or region of trial coordination office for the multi-country trials (n = 327)**

| **Country or region of multi-country trial coordination office** | Count (%) |
| --- | --- |
| North America* | 76 (23.2) |
| South America | 7 (2.1) |
| Europe | 48 (14.7) |
| Asia | 5 (1.5) |
| Australia | 2 (0.6) |
| Unknown or not reported | 189 (57.9) |

* included US and Canada

**Supplemental table 3.** Results from univariable logistic regression analysis for the relationship between trial characteristics and high-enrollment of BIPOC groups^1^

| **Trial factors** | **Overall** | **Black group** | **Hispanic group** | **Asian group** | **Other non-white groups** |
| --- | --- | --- | --- | --- | --- |
| Year of publication | | | | | |
| 2000-2009 | Ref | Ref | Ref | Ref | Ref |
| 2010-2020 | 1.45 (0.96, 2.21) | 0.65 (0.41, 1.03) | 0.86 (0.41, 1.83) | 3.00 (1.73, 5.20) | 1.11 (0.71, 1.74) |
| Sample size ≥ 716^*^ | 1.11 (0.75, 1.64) | 0.81 (0.53, 1.24) | 0.64 (0.30, 1.37) | 1.66 (1.05, 2.62) | 0.66 (0.44, 1.00) |
| Diabetes type | | | | | |
| Other | Ref | Ref | Ref | Ref | Ref |
| Type 2 diabetes | 1.21 (0.92, 1.60) | 0.94 (0.69, 1.28) | 1.12 (0.70, 1.79) | 2.36 (1.49, 3.73) | 1.19 (0.89, 1.59) |
| Enrollment location | | | | | |
| Other | Ref | Ref | Ref | Ref | Ref |
| Ambulatory | 0.72 (0.48, 1.07) | 1.23 (0.80, 1.89) | 1.00 (0.47, 2.12) | 0.48 (0.30, 0.79) | 1.03 (0.67, 1.57) |
| Random type | | | | | |
| Cluster | Ref | Ref | Ref | Ref | Ref |
| Individual | 0.51 (0.09, 2.80) | 0.51 (0.05, 5.67) | 2.04 (0.18, 23.15) | 2.03 (0.18, 22.61) | 1.57 (0.26, 9.50) |
| Trial primary objective | | | | | |
| Complication | Ref | Ref | Ref | Ref | Ref |
| Glucose control | 1.00 (0.59, 1.68) | 0.61 (0.34, 1.07) | 1.07 (0.44, 2.65) | 0.86 (0.46, 1.60) | 1.01 (0.58, 1.76) |
| Other | 1.01 (0.63, 1.65) | 0.67 (0.39, 1.15) | 1.82 (0.66, 5.04) | 1.36 (0.76, 2.44) | 0.98 (0.59, 1.64) |
| Intervention | | | | | |
| Type of intervention |  | | | | |
| Others | Ref | Ref | Ref | Ref | Ref |
| Medication | 0.69 (0.39, 1.20) | 0.30 (0.15, 0.60) | 1.10 (0.45, 2.71) | 3.36 (1.38, 8.16) | 0.96 (0.53, 1.72) |
| Frequency of intervention |  | | | | |
| Others | Ref | Ref | Ref | Ref | Ref |
| Weekly/daily | 0.51 (0.31, 0.85) | 0.58 (0.33, 1.00) | 1.00 (0.44, 2.28) | 1.49 (0.80, 2.75) | 1.17 (0.70, 1.98) |
| Duration of intervention > 6.5 month^*^ | 0.78 (0.51 1.18) | 1.45 (0.93, 2.27) | 0.56 (0.25, 1.25) | 0.92 (0.50, 1.50) | 1.06 (0.68, 1.64) |
| Follow-up | | | | | |
| Type of follow-up |  | | | | |
| Others | Ref | Ref | Ref | Ref | Ref |
| Face-to-face | 1.31 (0.69, 2.51) | 0.76 (0. 50, 1.17) | 0.68 (0.32, 1.47) | 1.81 (1.12, 2.93) | 1.20 (0.79, 1.82) |
| Frequency of follow-up |  | | | | |
| Other | Ref | Ref | Ref | Ref | Ref |
| Weekly/ Monthly | 1.09 (0.56, 2.13) | 1.61 (0.76, 3.44) | 0.26 (0.05, 1.31) | 0.46 (0.20, 1.07) | 0.84 (0.40, 1.73) |
| Duration of follow-up > 12 months^*^ | 1.24 (0.82, 1.87) | 1.52 (0.98, 2.38) | 0.89 (0.34, 2.30) | 0.90 (0.56, 1.44) | 1.20 (0.78, 1.86) |
| Funding source | | | | | |
| Non-industry | Ref | Ref | Ref | Ref | Ref |
| Industry | 0.77 (0.49, 1.21) | 0.53 (0.32, 0.89) | 1.17 (0.53, 2.58) | 1.83 (1.02, 3.27) | 0.80 (0.49, 1.30) |

* The cut-off point was determined by using the median value

^1^ Results shown as odds ratio (95% confidence interval), p-value

**Supplemental table 4.** Results from univariable linear regression analysis for the relationship between trial characteristics and enrollment rate of BIPOC groups^1^

| **Trial factors** | **Overall** | | **Black group** | | **Hispanic group** | | **Asian group** | | **Other non-white groups** | |
| --- | --- | --- | --- | --- | --- | --- | --- | --- | --- | --- |
| Year of publication | | | | | | | | | | |
| 2000-2009 | Ref | - | Ref | - | Ref | - | Ref | - | Ref | - |
| 2010-2020 | 4.84 (1.47, 8.22) | 0.005 | 0.31 (-1.62, 2.24) | 0.752 | 0.46 (-2.78, 3.71) | 0.777 | 5.12 (1.23, 9.02) | 0.010 | 0.75 (-0.96, 2.45) | 0.390 |
| Sample size ≥ 716^*^ | -1.00 (-4.21, 2.20) | 0.538 | -0.98 (-2.78, 0.81) | 0.281 | -0.81 (-4.06, 2.44) | 0.623 | 1.95 (-1.52, 5.41) | 0.269 | -2.05 (-3.61, -0.50) | 0.010 |
| Diabetes type | | | | | | | | | | |
| Other | Ref | - | Ref | - | Ref | - | Ref | - | Ref | - |
| T2DM | 1.67 (-0.60, 3.93) | 0.149 | -1.38 (-2.68, -0.09) | 0.036 | -0.68 (-2.71, -1.34) | 0.504 | 4.64 (1.97, 7.30) | 0.001 | 0.56 (-0.53, 1.65) | 0.313 |
| Enrollment location | | | | | | | | | | |
| Other | Ref | - | Ref | - | Ref | - | Ref | - | Ref | - |
| Ambulatory | -3.85 (-7.09, -0.61) | 0.020 | -1.04 (-2.87, 0.78) | 0.259 | -2.47 (-5.69, 0.76) | 0.133 | -3.30 (-6.90, 0.30) | 0.072 | -0.09 (-1.70, 1.51) | 0.909 |
| Random type | | | | | | | | | | |
| Cluster | Ref | - | Ref | - | Ref | - | Ref | - | Ref | - |
| Individual | -8.43 (-21.67, 4.81) | 0.211 | -2.27 (-11.88, 7.34) | 0.643 | 1.59 (-8.37, 11.55) | 0.753 | -12.74 (-29.98, 4.50) | 0.147 | -4.49 (-11.20, 2.21) | 0.188 |
| Trial primary objective | | | | | | | | | | |
| Complication | Ref | - | Ref | - | Ref | - | Ref | - | Ref | - |
| Glucose control | 0.46 (-3.80, 4.73) | 0.831 | -3.10 (-5.48, -0.73) | 0.011 | 0.42 (-3.39, 4.24) | 0.827 | 0.60 (-5.35, 4.16) | 0.806 | 1.14 (-0.96, 3.25) | 0.286 |
| Other | 1.89 (-2.09, 5.87) | 0.351 | -1.80 (-4.03, 0.45) | 0.117 | 4.78 (0.52, 9.03) | 0.028 | 1.66 (-2.75, 6.07) | 0.459 | 1.10 (-0.86, 3.06) | 0.269 |
| Intervention | | | | | | | | | | |
| Type of intervention |  | | | | | | | | | |
| Others | Ref | - | Ref | - | Ref | - | Ref | - | Ref | - |
| Medication | -3.00 (-8.56, 1.56) | 0.197 | -8.56 (-11.00, -6.13) | <0.001 | -2.22 (-6.07, 1.63) | 0.255 | 5.35 (-0.54, 11.24) | 0.075 | 0.31 (-1.92, 2.55) | 0.786 |
| Frequency of intervention |  | | | | | | | | | |
| Others | Ref | - | Ref | - | Ref | - | Ref | - | Ref | - |
| Weekly/daily | -2.42 (-6.44, 1.59) | 0.236 | -5.65 (-7.85, -3.45) | <0.001 | -1.39 (-4.96, -2.17) | 0.440 | 2.55 (-2.07, 7.16) | 0.279 | 1.15 (-0.85, 3.14) | 0.259 |
| Duration of intervention > 6.5 month^*^ | -4.00 (-7.38, -0.62) | 0.020 | 1.78 (-0.09, 3.66) | 0.062 | 0.83 (-4.26, 2.61) | 0.634 | -5.83 (-9.44, -2.21) | 0.002 | -0.83 (-2.50, 0.83) | 0.326 |
| Follow-up | | | | | | | | | | |
| Type of follow-up |  | | | | | | | | | |
| Others | Ref | - | Ref | - | Ref | - | Ref | - | Ref | - |
| Face-to-face | 0.33 (-2.91, 3.56) | 0.843 | -0.65 (-2.46, 1.16) | 0.481 | -0.36 (-3.69, 2.96) | 0.828 | 1.53 (-2.06, 5.12) | 0.402 | -0.29 (-1.88, 1.29) | 0.716 |
| Frequency of follow-up |  | | | | | | | | | |
| Other | Ref | - | Ref | - | Ref | - | Ref | - | Ref | - |
| Weekly/ Monthly | -0.32 (-5.81, 5.18) | 0.910 | 1.77 (-1.35, 4.89) | 0.265 | -3.00 (-8.89, 2.91) | 0.317 | -1.65 (-7.67, 4.37) | 0.590 | -0.45 (-3.22, 2.31) | 0.748 |
| Duration of follow-up > 12 months^*^ | -1.93 (-5.31, 1.46) | 0.264 | 1.13 (-0.75, 3.00) | 0.238 | -2.60 (-6.70, 1.50) | 0.211 | -3.2 (-6.80, 0.32) | 0.074 | -0.11 (-1.78, 1.55) | 0.894 |
| Funding source | | | | | | | | | | |
| Non-industry | Ref | - | Ref | - | Ref | - | Ref | - | Ref | - |
| Industry | -0.65 (-4.35, 3.05) | 0.729 | -5.42 (-7.46, -3.39) | 0.000 | -1.27 (-4.67, 2.14) | 0.463 | 4.96 (0.68, 9.23) | 0.023 | -0.21 (-2.06, 1.64) | 0.822 |

^*^ The cut-off point was determined by using the median value

^1^ Results shown as beta coefficients (95% confidence interval), p-value

**Supplemental table 5.** Results from multivariable linear regression analysis for the relationship between trial characteristics and enrollment rate of BIPOC groups^1^

| **Trial factors** | **Black group** | | **Hispanic group** | | **Asian group** | | **Other non-white groups** | |
| --- | --- | --- | --- | --- | --- | --- | --- | --- |
| Year of publication | | | | | | | | |
| 2000-2009 | Ref | - | Ref | - | Ref | - | Ref | - |
| 2010-2020 | -0.40 (-2.91, 2.11) | 0.755 | -1.74 (-6.05, 2.57) | 0.426 | 5.31 (0.12, 10.50) | 0.045 | 1.74 (-0.67, 4.16) | 0.157 |
| Sample size ≥ 716^*^ | -2.29 (-4.04, -0.54) | 0.010 | -0.62 (-4.38, 3.14) | 0.743 | 1.40 (-2.14, 4.94) | 0.437 | -2.29 (-3.95, -0.63) | 0.007 |
| Diabetes type | | | | | | | | |
| Other | Ref | - | Ref | - | Ref | - | Ref | - |
| T2DM | 0.26 (-1.04, 1.57) | 0.693 | -0.43 (-2.80, 1.94) | 0.721 | 4.92 (2.05, 7.79) | 0.001 | 0.65 (-0.54, 1.84) | 0.282 |
| Enrollment location | | | | | | | | |
| Other | Ref | - | Ref | - | Ref | - | Ref | - |
| Ambulatory | -2.47 (-5.42, 0.47) | 0.099 | -2.13 (-7.40, 3.14) | 0.424 | -1.09 (-7.14, 4.97) | 0.724 | 0.12 (-2.68, 2.92) | 0.933 |
| Random type | | | | | | | | |
| Cluster | Ref | - | Ref | - | Ref | - | Ref | - |
| Individual | 7.60 (-1.90, 17.09) | 0.116 | 7.49 (-3.60, 18.58) | 0.183 | -16.61 (-34.93, 1.70) | 0.075 | -6.39 (-13.65, 0.88) | 0.085 |
| Trial primary objective | | | | | | | | |
| Complication | Ref | - | Ref | - | Ref | - | Ref | - |
| Glucose control | -1.42 (-4.17, 1.34) | 0.311 | -0.35 (-5.52, 4.82) | 0.893 | -0.41 (-6.33, 5.51) | 0.891 | 0.43 (-2.20, 3.05) | 0.750 |
| Other | -2.26 (-4.70, 0.18) | 0.070 | 4.54 (-0.81, 9.89) | 0.095 | -1.17 (-6.05, 3.71) | 0.636 | 0.61 (-1.64, 2.86) | 0.593 |
| Intervention | | | | | | | | |
| Type of intervention |  | | | | | | | |
| Others | Ref | - | Ref | - | Ref | - | Ref | - |
| Medication | -7.47 (-10.58, -4.36) | <0.001 | -1.92 (-7.28, 3.44) | 0.478 | 2.86 (-4.28, 10.00) | 0.431 | 0.54 (-2.40, 3.49) | 0.717 |
| Frequency of intervention |  | | | | | | | |
| Others | Ref | - | Ref | - | Ref | - | Ref | - |
| Weekly/daily | -2.17 (-4.66, 0.32) | 0.087 | -1.11 (-5.74, 3.52) | 0.635 | -0.29 (-5.40, 4.83) | 0.912 | 1.28 (-1.15, 3.70) | 0.301 |
| Duration of intervention > 6.5 month^*^ | 0.91 (-0.98, 2.81) | 0.343 | -1.63 (-5.82, 2.57) | 0.444 | -5.97 (-9.78, -2.16) | 0.002 | -0.69 (-2.51, 1.14) | 0.460 |
| Follow-up | | | | | | | | |
| Type of follow-up |  | | | | | | | |
| Others | Ref | - | Ref | - | Ref | - | Ref | - |
| Face-to-face | -0.78 (-2.96, 1.40) | 0.484 | -2.23 (-6.18, 1.71) | 0.264 | -0.24 (-4.82, 4.34) | 0.918 | -0.49 (-2.63, 1.65) | 0.651 |
| Frequency of follow-up |  | | | | | | | |
| Other | Ref | - | Ref | - | Ref | - | Ref | - |
| Weekly/ Monthly | 1.69 (-1.27, 4.64) | 0.262 | -4.31 (-10.38, 1.75) | 0.161 | -2.11 (-8.02, 3.80) | 0.483 | -0.10 (-2.96, 2.75) | 0.943 |
| Duration of follow-up > 12 months^*^ | -0.21 (-2.22, 1.79) | 0.835 | -3.28 (-8.24, 1.69) | 0.193 | -0.59 (-4.51, 3.33) | 0.767 | 0.74 (-1.21, 2.68) | 0.456 |
| Funding source | | | | | | | | |
| Non-industry | Ref | - | Ref | - | Ref | - | Ref | - |
| Industry | -1.80 (-4.18, 0.57) | 0.136 | 0.25 (-4.57, 4.07) | 0.909 | 1.64 (-3.41, 6.69) | 0.524 | -1.11 (-3.33, 1.10) | 0.323 |

^*^ The cut-off point was determined by using the median value

^1^ Results shown as beta coefficients (95% confidence interval), p-value
